# Levels of type XVII collagen (BP180) ectodomain are elevated in circulation from patients with multiple cancer types and is prognostic for patients with metastatic colorectal cancer

**DOI:** 10.1186/s12885-023-11470-5

**Published:** 2023-10-06

**Authors:** Marina Crespo-Bravo, Jeppe Thorlacius-Ussing, Neel I. Nissen, Rasmus S. Pedersen, Mogens K. Boisen, Maria Liljefors, Astrid Z. Johansen, Julia S. Johansen, Morten A. Karsdal, Nicholas Willumsen

**Affiliations:** 1 Nordic Bioscience A/S, Herlev, 2730 Denmark; 2 Department of Biomedical Sciences, University of Copenhagen, Copenhagen, 2200 Denmark; 3 Department of Oncology, Copenhagen University Hospital – Herlev and Gentofte, Herlev, 2730 Denmark; 4 Department of Clinical Science, Intervention and Technology, Karolinska University Hospital Huddinge, Stockholm, 141 57 Sweden; 5 Department of Medicine, Copenhagen University Hospital – Herlev and Gentofte, Herlev, Copenhagen, 2730, 2900 Denmark; 6 Department of Clinical Medicine, Faculty of Health and Medical Sciences, University of Copenhagen, Copenhagen, 2200 Denmark

**Keywords:** Collagens, ECM, Non-invasive biomarker, Epithelial damage, Type XVII collagen, Ectodomain, mCRC, MACITs

## Abstract

**Background:**

Collagens are the major components of the extracellular matrix (ECM) and are known to contribute to tumor progression and metastasis. There are 28 different types of collagens each with unique functions in maintaining tissue structure and function. Type XVII collagen (BP180) is a type II transmembrane protein that provides stable adhesion between epithelial cells and the underlying basement membrane. Aberrant expression and ectodomain shedding of type XVII collagen have been associated with epithelial damage, tumor invasiveness, and metastasis in multiple tumor types and may consequently be used as a potential (non-invasive) biomarker in cancer and treatment target.

**Method:**

An ELISA targeting the type XVII collagen ectodomain (PRO-C17) was developed for use in serum. PRO-C17 was measured in a cohort of patients with 11 different cancer types (n = 214) and compared to healthy controls (n = 23) (cohort 1). Based on the findings from cohort 1, PRO-C17 and its association with survival was explored in patients with metastatic colorectal cancer (mCRC) treated with bevacizumab in combination with chemotherapy (n = 212) (cohort 2).

**Results:**

PRO-C17 was robust and specific towards the ectodomain of type XVII collagen. In cohort 1, PRO-C17 levels were elevated (*p* < 0.05) in serum from patients with CRC, kidney, ovarian, bladder, breast, and head and neck cancer compared to healthy controls. PRO-C17 was especially good at discriminating between CRC patients and healthy controls with an AUROC of 0.904. In cohort 2, patients with mCRC and high levels (tertile 3) of PRO-C17 had shorter overall survival (OS) with a median OS of 390 days compared to 539 days for patients with low levels of PRO-C17. When evaluated by multivariate Cox regression analysis, high PRO-C17 was predictive for poor OS independent of risk factors and the tumor fibrosis biomarker PRO-C3.

**Conclusion:**

PRO-C17 measures the ectodomain of type XVII collagen in serum and is a promising non-invasive biomarker that can aid in understanding tumor heterogeneity as well as elaborate on the role of collagen XVII in tumor progression. Moreover, the findings in the study proposes PRO-C17 as novel biomarker of epithelial damage in specific cancer types including CRC.

**Supplementary Information:**

The online version contains supplementary material available at 10.1186/s12885-023-11470-5.

## Introduction

Cancer is a major global health issue, and cancer incidence and mortality are increasing [1]. Biomarkers are critical for early diagnosis as well as to choose an appropriate treatment to reduce the burden of cancer [1]. In recent years, biomarker discovery efforts have been drawn to the tumor microenvironment (TME) and the extracellular matrix (ECM) that, when deregulated in cancer, can facilitate tumor progression and lack of response to cancer treatment [2]. Collagens are the main component of the ECM and to date, 28 different collagens have been identified [3]. In healthy tissue, collagen production and degradation are highly regulated processes aiming at maintaining tissue homeostasis. In cancer, however, excessive collagen turnover and remodeling occurs, resulting in loss of tissue organization and cellular behavior leading to disease progression [4].

Protein fragments that result from this excessive collagen remodeling are released into circulation and can be measured non-invasively in a liquid biopsy and used as biomarkers. For instance, high levels of type III collagen fragments (PRO-C3) have been associated with tumor fibrosis and poor overall survival in multiple cancer types such as pancreatic, colorectal, melanoma, liver, and breast cancer [5]. Even though extensive research and biomarker discovery have been directed towards the most predominant collagens of the ECM, such as type I, III, and IV, little is known about the less abundant, highly specialized group of collagens.

Membrane-associated collagens with interrupted triple-helices (MACITs), including type XIII, XVII, XXIII, and XXV, are type II proteins that contain large ectodomains not present in other collagens that can be shed and released into the bloodstream [6] [7]. Overall MACITs are expressed in low quantities in adult tissue but are upregulated during embryogenesis and tumorigenesis [8]. Even though MACITs can contribute to maintaining ECM stability, growing evidence demonstrates that they play essential roles in cell fate processes by binding to receptors or growth factors in healthy and cancerous tissue [9–11]. MACITs consist of an N-terminal cytoplasmatic domain, a transmembrane domain, and a C-terminal ectodomain includin different collagenous domains (COL) and non-collagenous domains (NC) [12]. Collagens XIII, XXIII, and XXV are similar in structure. Their ectodomain is cleaved by furin proteases, whereas collagen XVII differs structurally and is cleaved by ADAM 9 and 10 [13–15].

Type XVII collagen, also known as BP180 or BPAG2, consists of three identical 180-kDa α-chains and is part of the hemidesmosomes, providing stable adhesion between the basal keratinocytes and the basement membrane, thus playing a crucial role in the skin [16]. Several blistering skin diseases have been associated with genetic and acquired dysfunctions of type XVII collagen. For example, mutations in the *COL17A1* gene can cause junctional epidermolysis bullosa (JEB). Type XVII ectodomain cleavage occurs at different sites in the NC16A domain originating a 120 kDa fragment known as LAD-1, and autoimmunity towards this shed fragment of type XVII collagen induces bullous pemphigoid (BP) [17, 18]. LAD-1 can be further cleaved at the C-terminal region and release a 97 kDa polypeptide (LABD-97) associated with the development of the basement membrane [19, 20]. Moreover, LABD autoantibodies preferably react to LABD-97 than to LAD-1 leading to linear IgA bullous disorder (LABD) [21–23].

In cancer, type XVII collagen and its shed ectodomain appear to play a major role in epithelial cancer development and metastasis [24]. Abnormal expression of the *COL17A1* gene has been detected in many epithelial tumors such as colorectal (CRC), pancreatic, breast, cervical or squamous cell carcinoma and associated with poor prognosis [25–30]. Given the relevance of type XVII in tumorigenesis the development of a liquid biopsy biomarker targeting its ectodomain could not only provide valuable insights into the role of type XVII collagen in tumor progression but may also have potential as a non-invasive biomarker and diagnostic tool in cancer.

In this study, we developed and validated an ELISA targeting type XVII collagen ectodomain (PRO-C17) and measured the levels in serum samples to study its biomarker potential in patients with cancer. We found that PRO-C17 levels were significantly elevated in patients with different cancer types, and high levels of PRO-C17 were associated with lower OS in patients with mCRC.

## Materials and methods

### Antibody development for PRO-C17

A 10 amino-acid peptide ^524^LQGMAPAAGA^533^ corresponding to an internal epitope of the NC16A domain of type XVII collagen (UniProtKB: Q9UMD9) was purchased from Genscript (Piscataway, NJ, USA) and used for immunization. The immunogenic peptide (LQGMAPAAGA-GGC) was produced by binding the target peptide to keyhole limpet hemocyanin (KLH) carrier protein through a covalent cross-linking process using sulfosuccinimidyl 4-(N-maleimidomethyl) cyclohexane-1-carboxylate, SMCC (Thermo Scientific, Waltham, MA, USA, cat.no. 22,322). To ensure proper binding of the carrier protein, glycine and cysteine residues were incorporated at the N-terminal end. To generate monoclonal antibodies, six-week-old Balb/C mice were immunized subcutaneously with 200 µL of an emulsified antigen. The antigen consisted of 100 µg of immunogenic peptide mixed with Sigma Adjuvant System (Sigma cat. No. S6322) Specol (Invitrogen cat.No. 7,925,000). The mice were immunized repeatedly at 2-week intervals until a stable level of serum titer was achieved. The mouse with the highest titer was rested for four weeks and then received a boost of 100 µg of the immunogenic peptide in 100 µL of 0.9% NaCl solution through intravenous injection. Hybridoma cells were created by fusing spleen cells with SP2/0 myeloma cells using the method previously described [31]. The hybridoma cells were then cultured in 96-well microtiter plates, and standard limited dilution was used to secure monoclonal growth.

The monoclonal antibodies were purified using protein-G-columns according to the manufacturer’s instructions (GE Healthcare Life Sciences, Little Chalfont, UK, cat. #17-0404-01).

The antibody clone that was selected for the PRO-C17 ELISA development was based on a preliminary competitive ELISA testing for the reactivity towards the target peptide (LQGMAPAAGA) relative to an elongated peptide (RLQGMAPAAGA), a truncated peptide (QGMAPAAGA), two de-selection peptides (LQGMAYTVQG and LQGLAPLGSE), a target KLH-conjugated peptide (LQGMAPAAGA-GGC-KLH) and a non-sense KLH-conjugated peptide (DCTSTFPRV-GGC-KLH). Conditions were evaluated with different assay buffers, incubation time, and temperature as well as concentrations of antibodies and peptides.

### Technical evaluation of the PRO-C17 ELISA

The specificity of the antibody towards the target peptide (LQGMAPAAGA) was assessed by including elongated (RLQGCMAPAAGA) and truncated (QGMAPPAGA) versions of the peptide, as well as two deselection peptides corresponding to two proteins found in the extracellular matrix (collagen alpha-1 (XVII) chain: LQGMAYTVQG, and laminin subunit beta-4: LQGLAPLGSE) that have a similar sequence compared to the target peptide and could interfere with antibody binding. Linearity was validated by performing serial dilutions of serum samples in assay buffer and calculating the percentage recovery relative to the dilution. Accuracy of the assay was evaluated by spiking a serum sample with high concentration of the endogenous analyte with another serum sample with low concentration at different ratios (100:0, 75:25, 50:50, 25:75, 0:100) and calculating the percentage recovery relative to the analyte concentrations when the samples were measured separately. Interference towards the most common endogenous analytes (hemoglobin, biotin and lipids) was evaluated by spiking serum samples with known amounts of the interfering substances (hemoglobin low = 2.5 mg/mL, high = 5 mg/mL; lipids low = 1.5 mg/mL, high = 5 mg/mL; biotin low = 5 ng/mL, high = 100 ng/mL) and calculating the percentage of recovery compared to the non-spiked sample. The variation was determined by running 10 independent plates including 3 human serum samples, 3 quality control samples (QCs), 2 peptides in assay buffer and 2 kit controls (COs) in double determinations. Intra-assay variation was calculated as the mean CV% (coefficient of variation) between the sample replicates within the same plate. Intra variation was accepted at CV% <10. Inter-assay variation was calculated as the mean CV% between runs of sample replicates on different plates and the acceptance criteria was CV% <15%. Lower and upper limit of measurement range (LLOQ and ULOQ) were defined as the minimum concentrations where the mean CV% of serum samples was below 20%. Analyte stability was assessed by storing three serum samples for 2 h, 4 h, 24 h, and 48 h at both 4^o^C and 20^o^C and calculating the percentage of recovery compared to the corresponding control samples stored at -20^o^C. Freeze-thaw stability was determined by freezing and thawing serum samples for 5 cycles and calculating the percentage of recovery compared to the control samples only thawed once prior to be measured.

### PRO-C17 ELISA protocol

The final PRO-C17 protocol was the following: a 96-well streptavidin-coated ELISA plate was coated with 100 uL/well of 2.5 ng/mL of biotinylated QGMAPAAGA peptide dissolved in assay buffer (50 mM PBS, 1% BSA (w/v), 0.018% bronidox (v/v), 0.1% Tween-20 (w/v), 8 g/L NaCl, pH 7.4) incubated with shaking (300 rpm) for 30 min at 20^o^C in darkness. After washing 5 times with washing buffer (25 mM Tris, 50 mM NaCl, pH 7.2), 20 µL/well of pre-diluted sample (1:2) was added in duplicates followed by 100 µL/well of 25 ng/mL HRP-labelled monoclonal antibody in assay buffer and incubated with shaking (300 rpm) for 20 h at 4^o^C in darkness. After a second washing cycle, 100 µL/well of TMB was added and incubated with shaking (300 rpm) for 15 min at 20 ^o^C in darkness. The reaction was stopped by adding 100 µL/well of 1% H_2_SO_4_. Absorbance was measured at 450 nm with 650 nm as a reference. The standard curve was generated by adding 20 µL/well of 30 ng/mL QGMAPAAGADLDKIGLHSDSQEELWMFVRK peptide serially diluted twofold and a four-parameter logistic (4PL) model was used to fit a curve.

### Cell culture of A-431 cells

A-431 are epithelial cells derived from a patient with epidermoid carcinoma that constitutively express and cleave type XVII collagen. A-431 cells were purchased from ATCC (Rockville, MD, USA). The cells were cultured in two different conditions: keratinocyte serum-free medium (KSFM; Gibco, United Kingdom) supplemented with human recombinant epidermal growth factor (rEGF), bovine pituitary extract (BPE) and 0.4 mM CaCl_2_, or Dulbecco’s Modified Eagle’s Medium (DMEM, Gibco, United Kingdom) supplemented with 10% of fetal bovine serum (FBS; Gibco, United Kingdom).

### Western blot of A-431 cells

Supernatant and cell lysate from A-431 cells were run on a NuPAGE 4–12% Bis-Tris gel (Invitrogen, Carlsbad, CA, USA) under reducing conditions using a NuPAGE MES SDS running buffer (Invitrogen). The proteins from the polyacrylamide gel were transferred to an iBlot nitrocellulose membrane (Life Technologies, Bengaluru, India) using an iBlot Dry blotting system (Life Technologies, Carlsbad, CA, USA). Next, the membrane was blocked for 60 min with 5% skim milk (Sigma-Aldrich, St. Louis, MO, USA) in TBST (Tris-buffered saline (TBS) with 0.1%, Tween-20). After, the membrane was incubated overnight with 0.001 mg/mL of the type XVII collagen monoclonal antibody (PRO-C17) at 4^o^C. The day after the membrane was washed with TBST three times for 10 min and incubated right after with the secondary antibody (HRP-labelled) (1:5000) for 1 h. The membrane was washed with TBST three times for 10 min and incubated with SuperSignal west femto maximum sensitivity substrate (Thermo Fisher Scientific, Waltham, MA, USA) for 1–5 min and the protein bands were visualized on a C-DiGit Blot Scanner (LI-COR Biosciences, Lincoln, NE, USA). After development, the membrane was washed with TBST for 15 min. Stripping of the PRO-C17 antibody was performed by incubating the membrane with 15mL of Restore Western Blot Stripping buffer (Thermo Fisher Scientific, Waltham, MA, USA) for 15 min. The membrane was then washed three times for 10 min with TBST to remove the stripping buffer and re-blocked for 1 h using 30 mL of 5% skim milk in TBST. Thereafter the membrane was incubated with 1:1000 of commercial type XVII collagen antibody (MyBioSource, San Diego, CA, USA) and proceeded with the steps described before.

### Patient samples

Cohort 1 consisted of 214 patients with cancer and 23 healthy controls. It included 19 samples of bladder, breast, and prostate cancer, 20 samples of CRC, head and neck, kidney, melanoma, ovarian, pancreatic, and stomach cancer, 17 samples of lung cancer, and 23 age-matched healthy controls. Serum samples from patients with cancer collected at baseline were purchased from Proteogenex (Los Angeles, CA, USA). A summary of the cohort 1 characteristics can be found in Table 1. Serum samples were collected after patients gave their informed consent, approved by the Russian Oncological Research Centre n.a. Blokhin RAMS (PG-ONC 2033/1) (Moscow, Russia) and the Western Institutional Review Board, Inc. (Puyallup, WA, USA) (WIRB®Protocol #20,161,665). All investigations were conducted in accordance with the Helsinki Declaration.

**Table 1:** Demographics of the cohort 1

Characteristic	Cancer, N = 214	Healthy, N = 23
**Diagnosis, n (%)**		
**Bladder cancer**	19 (8.9)	
**Breast cancer**	19 (8.9)	
**Colorectal cancer**	20 (9.3)	
**Head & neck cancer**	20 (9.3)	
**Kidney cancer**	20 (9.3)	
**Lung cancer**	17 (7.9)	
**Melanoma**	20 (9.3)	
**Ovarian cancer**	20 (9.3)	
**Pancreatic cancer**	20 (9.3)	
**Prostate cancer**	19 (8.9)	
**Stomach cancer**	20 (9.3)	
**Healthy donors**		23 (100)
**Stages, n (%)**		
**I**	7 (3.3)	
**II**	45 (21)	
**III**	89 (42)	
**IV**	73 (34)	
**Age, Mean (SD)**	59 (11)	57 (6)
**Sex, n (%)**		
**Male**	115 (54)	16 (70)
**Female**	99 (46)	7 (30)

Cohort 2 comprised serum samples from 212 patients with mCRC part of the study CREBB “ColoRectal cancer – Evaluation of Biomarkers in Bevacizumab treatment” from 2011 to 2016 from four Swedish and three Danish hospitals. Serum samples were collected at baseline before palliative treatment with chemotherapy combined with bevacizumab. The study was performed according to the recommendations of the Danish Regional Committee on Health Research Ethics. The CREBB protocol was approved by the Danish Regional Committee on Health Research Ethics (Approval ID: H-3-2010-121) and the Data Protection Agency (Approval ID: 2007-58-0015/HEH.750.24-44). The study was conducted in accordance with the principles of the Declaration of Helsinki and all patients provided written informed consent prior to enrolment. Clinical data included: Age, sex, total number of drugs in the treatment regimen, primary tumor location, number of metastatic sites at study inclusion, performance status, synchronous metastatic disease, information about primary tumor resection, and PRO-C3 levels at baseline. A summary of the cohort 2 characteristics can be found in Table 2. The serum samples from healthy controls used both in cohort 1 and 2 were obtained from BioIVT (Westbury, NY, USA).

**Table 2:** Demographics of the cohort 2

Characteristic	Healthy, N = 28	Cancer, N = 212
**Sex**		
**Female**	15 (54%)	85 (40%)
**Male**	13 (46%)	127 (60%)
**Age**		
**Median (IQR)**	52 (49, 57)	67 (60, 73)
**Range**	32, 72	31, 99
**Primary tumor location**		
**Right colon**		69 (33%)
**Left colon and rectum**		136 (64%)
**Appendix, small intestine**		7 (3.3%)
**Synchronous metastatic disease (Sync)**		
**No**		64 (30%)
**Yes**		148 (70%)
**Primary tumor resection (OP)**		
**Primary resected at baseline**		101 (48%)
**Primary in situ at baseline**		111 (52%)
**Performance status (PS)**		
**0**		134 (65%)
**1**		63 (31%)
**2**		7 (3.4%)
**3**		2 (1.0%)
**Unknown**		6
**Number of metastatic sites**		
**0**		3 (1.4%)
**1**		79 (37%)
**2**		95 (45%)
**3**		27 (13%)
**4**		8 (3.8%)
**Line of palliative chemotherapy**		
**1**		127 (60%)
**2**		69 (33%)
**3**		12 (5.7%)
**4**		2 (0.9%)
**Unknown**		2 (0.9%)
**Total drugs in treatment regimen**		
**2**		58 (27%)
**3**		151 (71%)
**4**		3 (1.4%)
**Serum CEA (µg/L)**		
**Median (IQR)**		26 (7, 93)
**Unknown**		67
**Leucocytes (10** ^**9**^ **/L)**		
**Median (IQR)**		7.00 (5.50, 8.80)
**Unknown**		5
**Platelets (10** ^**9**^ **/L)**		
**Median (IQR)**		270 (207, 332)
**Unknown**		5
**Neutrophils (10** ^**9**^ **/L)**		
**Median (IQR)**		4.37 (3.26, 6.00)
**Unknown**		55
**Lactate dehydrogenase (LDH) (U/L)**		
**Median (IQR)**		208 (176, 276)
**Unknown**		24
**PRO-C3 (ng/mL)**		
**Median (IQR)**		13 (11, 23)
**PRO-C17 (ng/mL)**		
**Median (IQR)**	1.53 (1.30, 1.86)	2.25 (1.80, 2.66)
**Unknown**	0	1

### Statistics

In cohort 1, PRO-C17 levels in patients with different cancer types were compared to the level in healthy controls by performing a one-way ANOVA followed by multiple comparisons using Dunn’s test. The diagnostic accuracy of PRO-C17 was assessed by calculating the area under the receiver operating characteristics (AUROC) curve and evaluating the ability to discriminate individual cancer types from healthy controls. In cohort 2, PRO-C17 levels in patients with mCRC were compared to PRO-C17 levels in healthy controls with a t-test. The association between PRO-C17 and OS was evaluated by dividing patients into those with low PRO-C17 levels (tertile 1 + tertile 2) and compare that to patients with high PRO-C17 levels (tertile 3). Kaplan-Meier curves and log-rank tests were applied to determine differences between the survival curves. Multivariate Cox regression analysis stratified by line of treatment was performed to evaluate the independent predictive value of PRO-C17 after adjusting for age, sex, total number of drugs, location, synchronous metastatic disease, performance status, primary tumor resection, number of metastatic sites and PRO-C3 levels at baseline. Significance was considered with *p*-values < 0.05 as it follows: * p < 0.05; ** p < 0.01; *** p < 0.001; **** p < 0.0001. Statistical analyses were performed using GraphPad Prism (version 9.5.0 for Windows, GraphPad Software, San Diego, California USA, www.graphpad.com) and R version 4.2.2 (R Core Team (2022), R Foundation for Statistical Computing, Vienna, Austria, https://www.R-project.org).

## Results

### PRO-C17 ELISA development

#### Epitope mapping and in vitro shedding of type XVII collagen ectodomain confirms specificity of PRO-C17

**Fig. 1:**
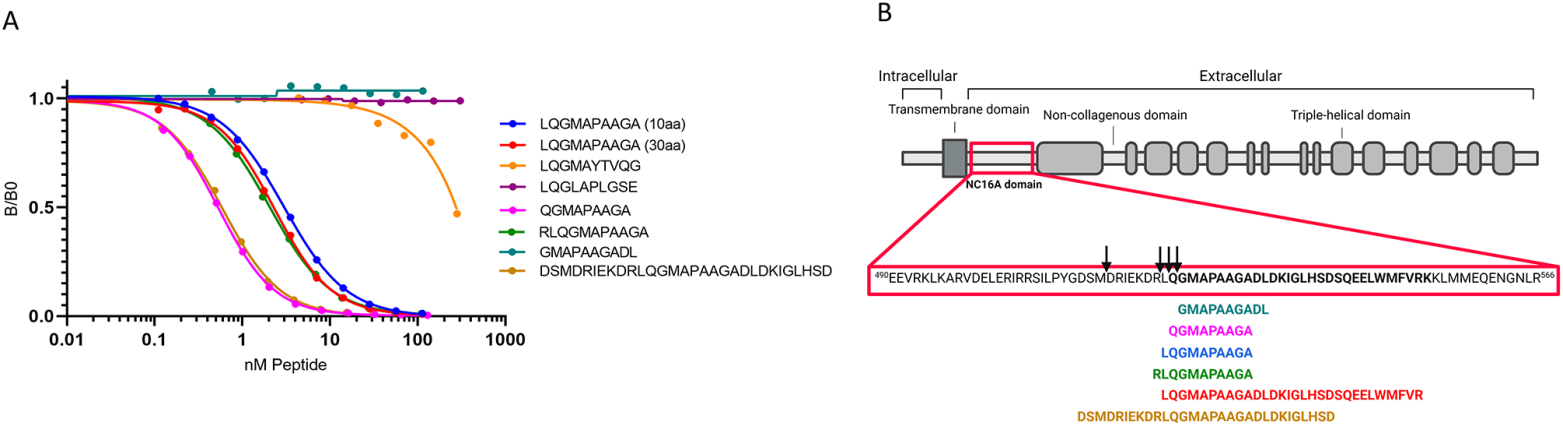
(**A**) Specificity and epitope mapping of the PRO-C17 monoclonal antibody. The monoclonal antibody’s reactivity in the competitive PRO-C17 ELISA was evaluated toward the 10aa target peptide (LQGMAPAAGA), 30aa version of the target peptide (LQGMAPAAGADLDKIGLHSDSQEELWMFVRK), two elongated peptides (RLQGCMAPAAGA and DSMDRIEKDRLQGMAPAAGADLDKIGLHSD), two truncated peptides (QGMAPAAGA and GMAPAAGADL) and two deselection peptides (LQGMAYTVQG and LQGLAPLGSE). The monoclonal antibody was also specific for the elongated peptide (RLQGCMAPAAGA) and the truncated peptide (QGMAPPAGA). B/B0 is the ratio between the OD when the analyte is present (B) and the maximum OD when the analyte is not present (B0). (**B**) NC16A domain of type XVII collagen. Physiological cleavage sites are indicated with black arrows and target peptide in blue

**Fig. 2:**
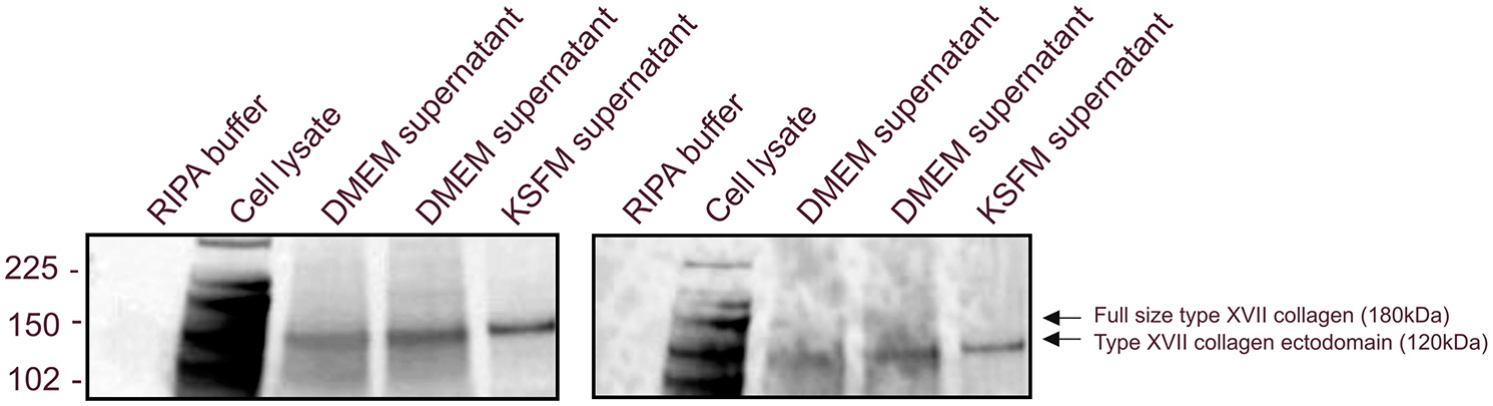
Western blot results of type XVII collagen in supernatant and cell lysate from A-431 cells with the commercial type XVII antibody (right) and the PRO-C17 antibody (left). In supernatant only the 120 kDa fragment corresponding to the type XVII ectodomain was detected in the cell supernatant. The 180 kDa fragment corresponding to the full size of type XVII collagen was detected only in the cell lysate. We used one replicate for the cell lysate, two replicates for the cell supernatant in DMEM media and one replicate for the cell supernatant in KSFM media. Uncropped versions of the western blot can be found in the supplementary material as Supplementary Figure S1

The specificity of the assay was assessed by the ability of different peptides to compete for binding to the monoclonal antibody in a competitive ELISA format (Fig. 1A and B). The epitope mapping showed that the antibody recognized both the 10 and 30 amino acid target peptides, one of the truncated (QGMAPAAGA) and both elongated peptides, whereas no cross-reactivity was observed towards the deselection peptides and the truncated peptide GMAPAAGADL, suggesting that the antibody is not neo-epitope specific but sequence specific (Fig. 1A). To further confirm that the PRO-C17 ELISA detects shedding of type XVII ectodomain in circulation, we performed a western blot on supernatant and cell lysate from A-431 cells. The PRO-C17 monoclonal antibody and a commercial type XVII collagen antibody were used for comparison. Type XVII collagen ectodomain was present in the A-431 supernatant revealing a 120 kDa fragment when using either of the two antibodies. As the western blot was conducted under denatured protein conditions the full-length type XVII collagen of 180 kDa was also detected, but only in the cell lysate (Fig. 2). These findings support that even though PRO-C17 is not neoepitope specific, the assay quantifies type XVII collagen ectodomain in circulation.

### Technical evaluation of the PRO-C17 ELISA assay

Intra- and inter-assay variations were 4% and 9% based on 10 independent runs, respectively. Linearity of dilution was accepted from 1:2 to 1:16. Matrix-in-matrix spiking test for accuracy testing showed a recovery of 99.6%. Interference from common endogenous analytes was within the accepted recovery rates (80–120%) for all samples. Analyte stability was evaluated for up to 48 h at 4^o^C or 20^o^C, with a mean recovery of 103% and 107% respectively. Stability after four freeze-thaw cycles had a 99% recovery rate. The different technical validation steps are summarized in Table 3.

**Table 3:** Summary of the technical validation of PRO-C17

Assay parameter	Result
**ELISA format**	Competitive ELISA with TMB
**Intended matrix (MRD)**	Human serum (1 + 1)
**Incubation buffer**	50mM PBS-BTB, 8 g/L NaCl
**Lower and upper limit of quantification (LLOQ-ULOQ)**	0.43-15 (ng/mL) uncorrected 0.86-30 (ng/mL) corrected
**Lower limit of blank (LLOB)**	0.33 ng/mL
**Mean slope**	1.1
**Mean IC25** **Mean IC50** **Mean IC75**	0.8 ng/mL 2.1 ng/mL 5.5 ng/mL
**Intra-assay coefficient of variation (CV%)**	4%
**Inter assay coefficient of variation (CV%)**	9.3%
**Dilution recovery (1 + 1 from MRD)**	103.7%
**Accepted maximum freeze-thaw**	99%
**Interference lipid, low/high**	102%/93%
**Interference hemoglobin, low/high**	96%/93%
**Interference biotin, low/high**	101%/107%
**Analyte stability (48 h 4 °C/48 h 20 °C)**	103%/107%

### PRO-C17 is elevated in patients with different types of cancer

**Fig. 3:**
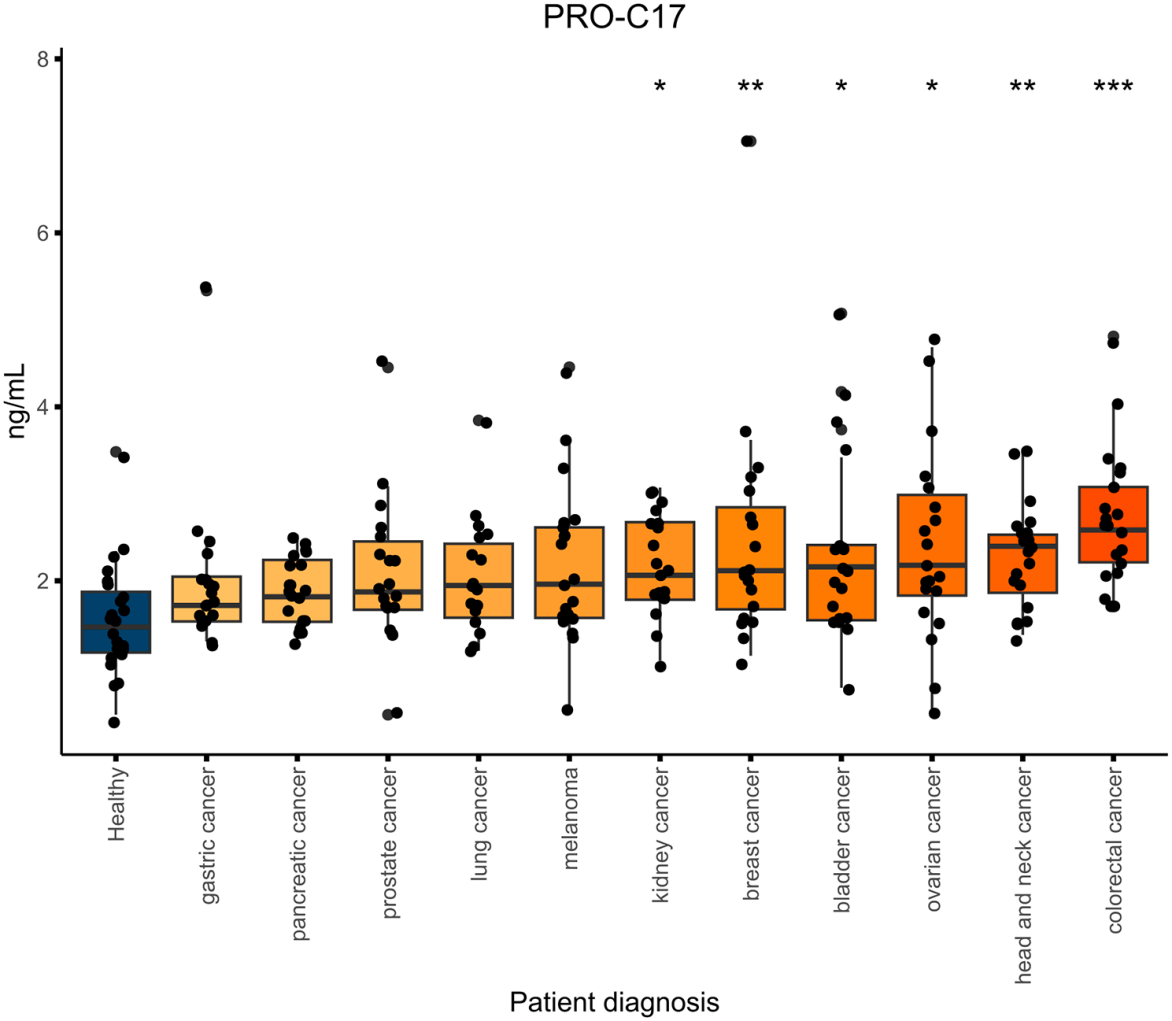
PRO-C17 in serum of cancer patients. Quantification of PRO-C17 in the serum of healthy controls (n = 23) and bladder cancer (n = 19), breast cancer (n = 19), colorectal cancer (n = 20), head & neck cancer (n = 20), kidney cancer (n = 20), lung cancer (n = 17), melanoma (n = 20), ovarian cancer (n = 20), pancreatic cancer (n = 20), prostate cancer (n = 19) and stomach cancer (n = 20). PRO-C17 levels are presented as Tukey-style boxplots with data-point jitter: horizontal bars indicate the median; upper- and lower hinges of the box indicate the first and third quartiles (the 25th and 75th percentiles); whiskers extend from the hinges to the largest or smallest value but no further than 1.5*IQR (where IQR is the inter-quartile range between the first and third quartiles) in either the positive or negative direction. Samples measuring below the LLOQ were given the value of LLOQ, as determined in the validation of PRO-C17. Differences in PRO-C17 levels between the cancer types and the healthy controls were evaluated by ordinary ANOVA followed by Dunn’s multiple comparisons t-test. **** indicates a *p* value below 0.0001. *** below 0.001. ** below 0.01. * below 0.05

To investigate the non-invasive biomarker potential of PRO-C17 in cancer, we measured PRO-C17 in a cohort consisting of 214 patients with different types of cancer (cohort 1, Table 1). We found elevated PRO-C17 levels in serum from patients with CRC, kidney-, ovarian-, bladder-, breast-, and head and neck cancer compared to healthy controls (p < 0.05) - but not in patients with melanoma, pancreatic-, lung-, prostate-, and gastric cancer (Fig. 3). After stratifying patients according to cancer stage, we did not find an association with the PRO-C17 levels, even though the biomarker levels seem to increase in late-stage patients in some cancer types (Supplementary Figure S2). The diagnostic accuracy of PRO-C17 to discriminate between healthy donors and cancer patients was illustrated by the AUROC (Table 4). PRO-C17 was especially good at discriminating between patients with CRC and healthy controls with an AUROC of 0.904 (Table 4). These results suggest that circulating fragments of type XVII collagen (PRO-C17) are elevated in several, but not all, cancer types.

**Table 4:** Area under the ROC curve (AUROC) for the comparison between cancers and controls

Cancer type	AUROC	Cutoff	Sensitivity	Specificity
**Colorectal cancer**	0.904	1.788	0.95	0.739
**Head & neck cancer**	0.828	2.005	0.7	0.826
**Kidney cancer**	0.789	1.643	0.9	0.652
**Breast cancer**	0.777	1.986	0.632	0.826
**Ovarian cancer**	0.759	1.829	0.75	0.739
**Bladder cancer**	0.752	1.994	0.632	0.826
**Lung cancer**	0.739	1.776	0.706	0.739
**Melanoma**	0.737	2.351	0.45	0.957
**Prostate cancer**	0.727	1.637	0.789	0.652
**Pancreatic cancer**	0.722	1.314	1	0.391
**Stomach cancer**	0.69	1.481	0.85	0.522

### PRO-C17 predicts poor outcome in patients with mCRC

**Fig. 4:**
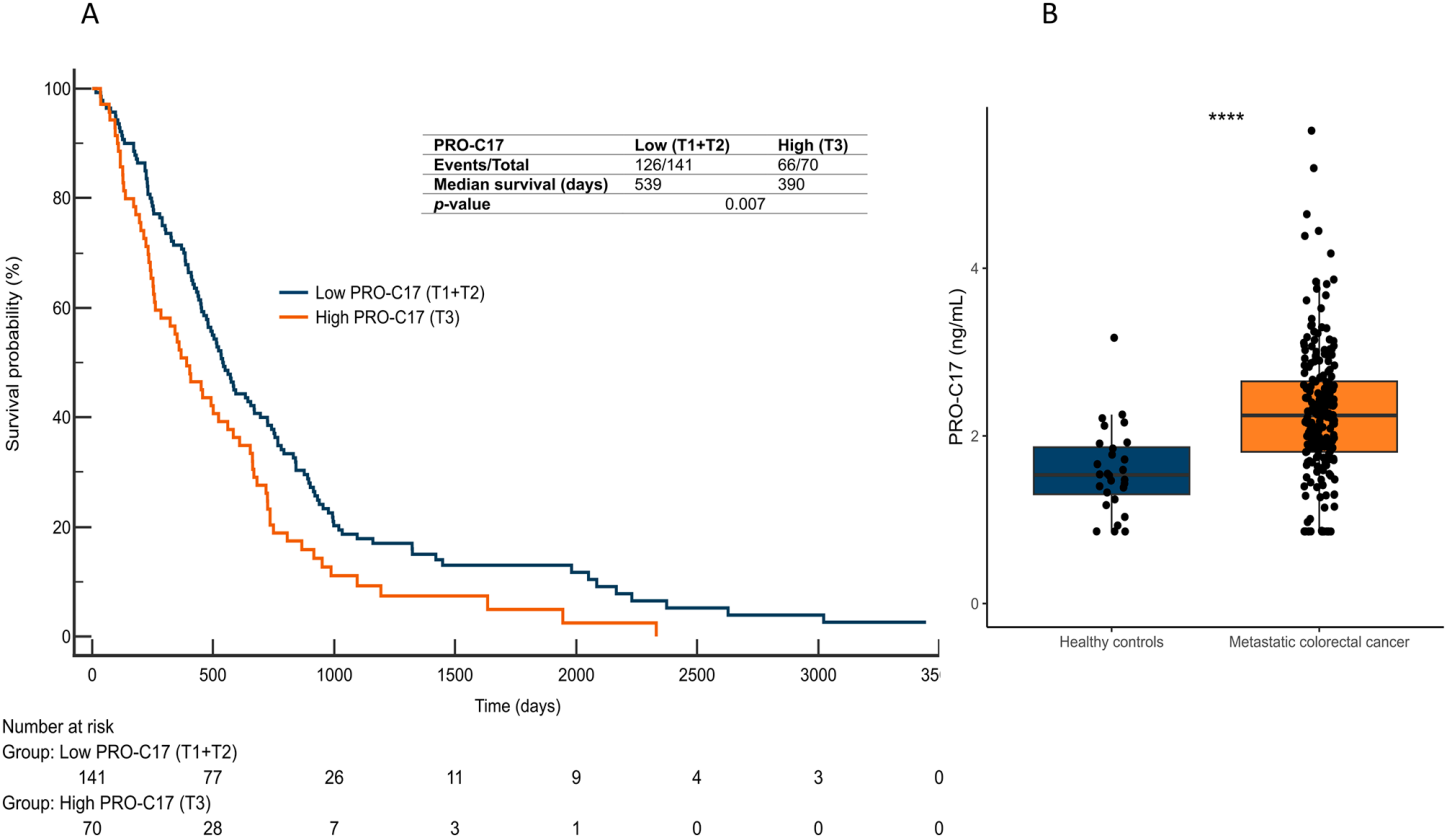
(**A**) PRO-C17 levels were significantly elevated in patients with metastatic colorectal cancer (n = 212) compared to healthy controls (n = 23). The comparison was performed with a parametric t-test. **** indicates *p* < 0.0001. (**B**) Kaplan-Meier survival plots showing that a high level of PRO-C17 was associated with shorter OS in patients with mCRC.

Based on the results observed in cohort 1 where patients with CRC had the highest PRO-C17 levels, we thereafter evaluated the potential use of PRO-C17 as a biomarker in patients with mCRC (cohort 2). PRO-C17 levels were increased in patients with mCRC compared to healthy subjects (*p* < 0.0001) (Fig. 4A), in line with our findings from cohort 1. Further, PRO-C17 levels in the healthy controls remained very similar with a median of 1.46 ng/mL and 1.53 ng/mL in cohorts 1 and 2, respectively. To investigate the prognostic value of PRO-C17, patients were dichotomized into two groups according to biomarker levels: “low” (tertile 1 + tertile 2) and “high” (tertile 3). The Kaplan-Meier survival analysis showed that patients with low PRO-C17 had a median OS of 539 days compared to the 390 days for patients with high PRO-C17 levels (log-rank, *p* = 0.007) (Fig. 4B). The ability of PRO-C17 to predict OS was then investigated with a multivariate Cox proportional-hazards model stratified by line of treatment and including the covariates age, sex, total number of drugs, synchronous metastatic disease, type III collagen pro-peptides (PRO-C3) levels in serum at baseline, location of the tumor, primary tumor resection, performance status and number of metastatic sites. High PRO-C17 level remained independently predictive of increased risk of dying (HR = 1.43, 95% CI 1.01–2.01, *p* = 0.044) (Supplementary Figure S3).

## Discussion

In the past, multiple studies have shown how type XVII collagen plays an important role in tumor development and invasiveness by looking at gene expression or immunostaining of type XVII collagen. For example, in patients with epithelial cancers such us breast and cervical cancer aberrant *COL17A1* promoter methylation is predictive of poor prognosis [27]. In skin cancers like squamous cell carcinoma (SCC), immunohistochemical analysis has shown a correlation between the presence of type XVII collagen in the primary tumor and metastasis and increased expression of *COL17A1* was associated with poor survival in patients with CRC [25, 32].

For the first time, in this study we assessed the collagen XVII ectodomain levels in serum by developing a PRO-C17 competitive ELISA. The assay was technically robust and capable of measuring the type XVII collagen ectodomain released into circulation in an accurate and specific manner. We found increased PRO-C17 levels in patients with different types of cancer (CRC, bladder-, breast-, head and neck-, kidney-, and ovarian cancer) compared to healthy individuals, but not in patients with melanoma, gastric-, lung-, pancreatic and prostate cancer. In head and neck cancer and particularly among in cancers originating from the larynx and the oral cavity, lower expression of *COL17A1* in early stages during cancer development may reflect alterations on keratinocytes binding to the ECM. On the contrary, higher expression of type XVII in patients with advanced cancer stages could be related to tumor progression [28]. Moreover, other studies suggest that type XVII collagen could have a chemotactic role when interacting with αII_b_ integrin in the tumor front and interestingly, in breast cancer cells type XVII collagen seems to suppress tumor growth by reducing the expression of Ki67 and deactivating the mTOR signaling pathway [33, 34]. Nevertheless, very few studies have shown a clear role of type XVII collagen in bladder and kidney cancer while overexpression of *COL17A1* has been detected in cervical tumors [27]. Patients with CRC had the highest PRO-C17 levels, and PRO-C17 was best at discriminating these patients from healthy controls. Given the relevance of type XVII in collagen CRC progression, we investigated the potential use of PRO-C17 as a prognostic biomarker in patients with mCRC prior to palliative treatment with the anti-angiogenic drug bevacizumab in combination with chemotherapy.

Bevacizumab, an anti-VEGF antibody, prevents the growth of new blood vessels in the tumor tissue, increases vascular permeability facilitating the delivery of chemotherapeutic drugs, and promotes apoptosis of tumor cells [35]. Additionally, treatment with bevacizumab has shown to improve OS in patients with mCRC [36, 37]. We observed increased PRO-C17 levels in patients with mCRC compared to healthy individuals. Moreover, we also found that high levels of PRO-C17 were associated with short OS, independently of other risk factors such as number of metastases, performance status, total amount of drugs, resection of the primary tumor and more interestingly, PRO-C3 levels.

PRO-C3 is a biomarker of cancer-associated fibroblast (CAF) activity reflecting type III collagen formation. The excessive collagen deposition and ECM remodeling during tumor fibrosis is associated with drug resistance, increased cell migration, and higher proliferation rate of cancer cells in vitro [38, 39]. PRO-C3 was previously assessed in these cohorts of patients and likewise, high levels of the biomarker correlated with poor survival [40]. Taken together, these results indicate that PRO-C17 and PRO-C3 correlate with different biological processes taking place in the ECM and provide additive prognostic value. Whereas PRO-C3 reflect the excessive ECM deposition in the TME, it could be argued that high levels of PRO-C17 may be result of the epithelial damage induced by the excessive remodeling of the ECM surrounding the tumor, characterized by the loss of cell adhesion molecules resulting in insufficient cell traction and therefore increased cell motility and metastasis [30]. Recently, alterations in *COL17A1* expression and aberrant ectodomain shedding have been reported to play a role in epithelial tumor development and cell migration [27, 28, 32, 33, 41]. In addition, type XVII collagen has been associated with tumor initiation, metastasis, and poor OS in patients with CRC [25, 42, 43]. In a recent study, *COL17A1*-knockout (KO) CRC organoids were generated to investigate the role of type XVII collagen in the maintenance of cancer stem cells (CSC) dormancy. *COL17A1*-KO CRC organoids expressed *LGR5* but lost the expression of *p27* in a similar way as it occurred in *COL17A1*-wild-type (WT) organoids, indicating that type XVII collagen regulated *p27* expression independently from *LGR5*. *COL17A1*-KO organoids were more susceptible to chemotherapy treatment, suggesting that chemotherapy induces ECM remodeling and subsequently type XVII ectodomain shedding that would initiate the proliferation of dormant cancer cells. Taken together, these studies indicate that type XVII collagen is essential for maintaining cancer cells in cell cycle arrest, but after treatment with chemotherapy and thus shedding of type XVII collagen, dormant cancer cells start proliferating through FAK-YAP activation leading to tumor regrowth [43]. In this context, PRO-C17 could provide valuable insights regarding potential cancer recurrence in patients treated with chemotherapy that could benefit from novel therapeutic strategies targeting YAP signaling.

In the past, dysfunctions in type XVII collagen have been associated with blistering skin diseases. For example, autoimmunity towards the ectodomain of type XVII collagen induces bullous pemphigoid [44, 45]. Moreover, in a study of patients with non-small cell lung cancer (NSCLC) treated with the immune checkpoint inhibitors (ICIs) anti-PD1/PD-L1, high levels of antibodies towards type XVII collagen and immune-related adverse events (irAEs) were associated with better response to therapy and longer OS [46]. It has not been investigated if there is a correlation between type XVII collagen and a beneficial response to ICIs, but as it occurs in patients with melanoma, type XVII collagen could have a crucial role in maintaining T-cell effector function during ICI therapy [47]. In this particular context, patients receiving PD-1 treatment could potentially have elevated levels of anti-BP180-antibodies and this raises the possibility of a competitive interaction between these native antibodies and those used in the PRO-C17 assay. Further studies are needed to explore this interaction. Nonetheless, these results encourage further research into the use of PRO-C17 to predict therapy response, as it could provide a tool for identifying patients with increased levels of autoimmunity towards type XVII collagen ectodomain that could potentially benefit from ICI in patients with NSCLC but also in patients with other cancer types where immunotherapy is a standard treatment option.

A limitation of our study is the size of cohort 1 as well as the paucity of clinical data and other risk factors that could provide valuable insights to the interpretation of our results. Additionally, it could be interesting to study the prognostic value of serum PRO-C17 levels in patients with early-stage cancer and to investigate if loss of type XVII ectodomain is driven by chemotherapy and correlates with cancer relapse by including on-treatment samples. Based on our results, serum PRO-C17 could be used as a biomarker to identify patients that are more prompted to cancer recurrence after chemotherapy treatment and provide a therapeutic approach to stop tumor regrowth.

## Conclusion

We successfully developed, optimized, and validated an ELISA to quantify type XVII collagen ectodomain shedding in serum (PRO-C17). The PRO-C17 ELISA is a technically robust, precise, and sensitive assay. PRO-C17 levels were increased in patients with different types of cancer, especially in CRC, when compared to levels in healthy controls. In patients with mCRC treated with bevacizumab in combination with chemotherapy, a high level of PRO-C17 was associated with short OS. This study suggests that serum PRO-C17 could be a potential biomarker of epithelial damage/remodeling in specific cancer types such as CRC and warrants further investigations.

### Electronic supplementary material

Below is the link to the electronic supplementary material.

Supplementary Material 1

## Data Availability

The data presented in this study can be available upon request and obtained from the corresponding author.

## Declarations

In cohort 1, serum samples were collected after patients gave their informed consent, approved by the Russian Oncological Research Centre n.a. Blokhin RAMS (PG-ONC 2033/1) (Moscow, Russia) and the Western Institutional Review Board, Inc. (Puyallup, WA, USA) (WIRB®Protocol #20161665). All investigations were conducted in accordance with the Helsinki Declaration.

In cohort 2, the study was performed according to the recommendations of the Danish Regional Committee on Health Research Ethics. The CREBB protocol was approved by the Danish Regional Committee on Health Research Ethics (Approval ID: H-3-2010-121) and the Data Protection Agency (Approval ID: 2007-58-0015/HEH.750.24-44). The study was conducted in accordance with the principles of the Declaration of Helsinki and all patients provided written informed consent prior to enrolment.

## Abbreviations

ECM: Extracellular Matrix
mCRC: metastatic colorectal cancer
OS: overall survival
HR: hazard ratio
TME: tumor microenvironment
MACITs: membrane-associated collagen with interrupted triple helices
COL: collagenous
NC: non-collagenous
JEB: junctional epidermolysis bullosa
BP: bullous pemphigoid
KLH: keyhole limpet hemocyanin
QC: quality control
CO: control
LLOQ: lower limit of quantification
ULOQ: upper limit of quantification
ELISA: enzyme-linked immunosorbent assay
PBS: phosphate-buffered saline
BSA: bovine serum albumin
Rpm: revolutions per minute
TMB: 3,3’,5,5’-Tetramethylbenzidine
HRP: horseradish peroxidase
AUROC: area under the receiver operating characteristic
CAF: cancer-associated fibroblast
NSCLC: non-small cell lung cancer
ICI: immune checkpoint inhibitor
irAE: immune-related adverse event
