# Supplementary material for: Levels of type XVII collagen (BP180) ectodomain are elevated in circulation from patients with multiple cancer types and is prognostic for patients with metastatic colorectal cancer
Source: BMC Cancer. 2023 Oct 6;23:949. doi: 10.1186/s12885-023-11470-5 (PMC10557271; doi:10.1186/s12885-023-11470-5)
Supplement: Supplementary file 1 — Supplementary Material 1 [file 12885_2023_11470_MOESM1_ESM.pdf]

Supplementary material

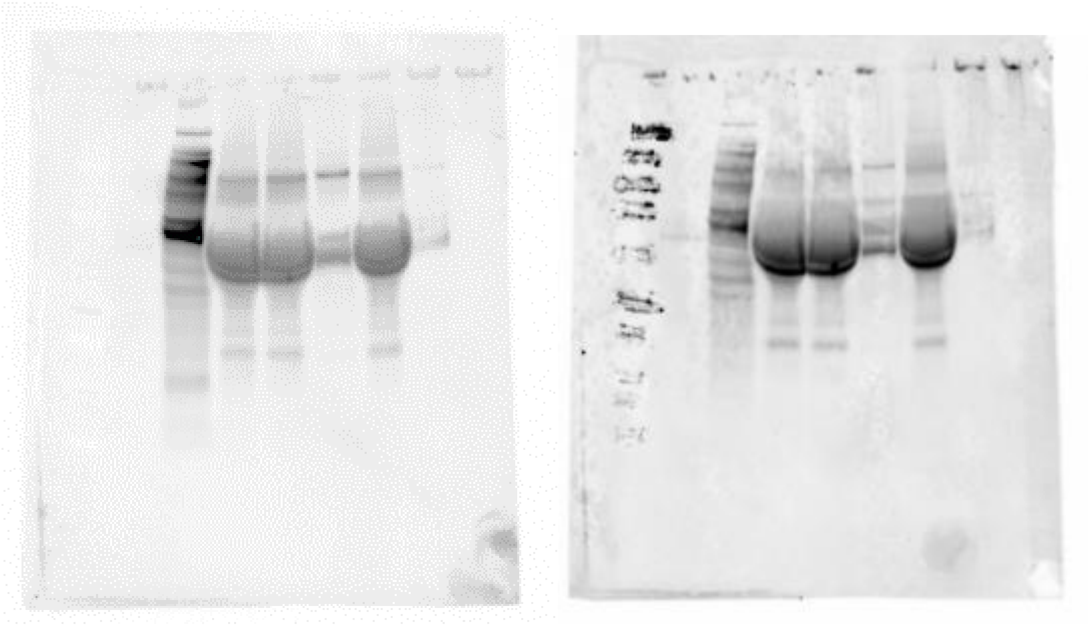

Supplementary Figure S1 Uncropped western blot results of type XVII collagen in supernatant and cell lysate from A-431 cells with the commercial type XVII antibody (right) and with the PRO-C17 antibody (left).

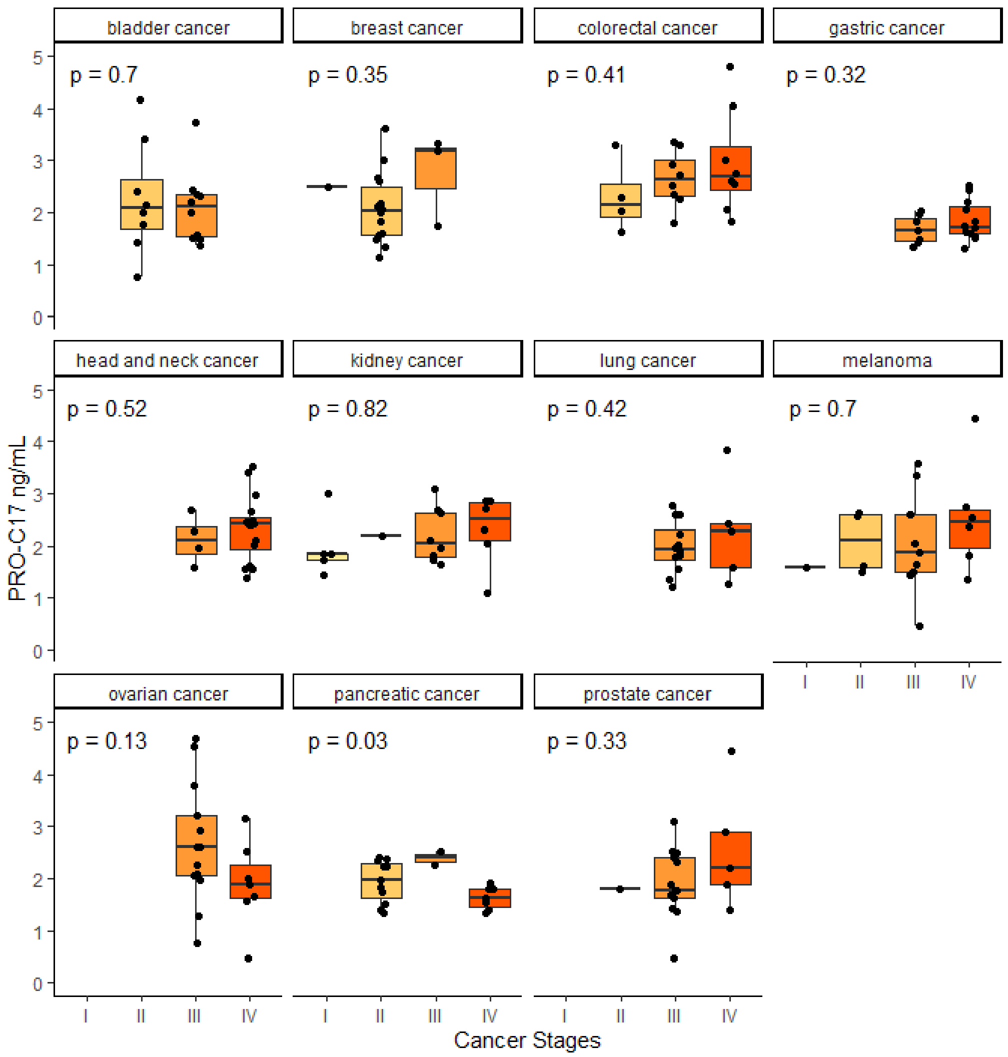

Supplementary Figure S2 PRO-C17 in cohort 1 according to cancer stage. PRO-C17 levels are displayed as Tukey-style boxplots with datapoint jitter. Differences in the biomarker levels in the different stages in each cancer type was analyzed with ANOVA.

Variable		N	Hazard ratio		p
PRO-C17	Low	132		Reference	
	High	64		1.43 (1.01, 2.01)	0.044
PRO-C3	Low	98		Reference	
	High	98		1.83 (1.30, 2.58)	<0.001
Total_drugs	2	50		Reference	
	3	143		1.09 (0.70, 1.69)	0.714
	4	3		0.40 (0.09, 1.74)	0.222
Age		196		1.02 (1.00, 1.04)	0.127
Sex	Male	118		Reference	
	Female	78		1.11 (0.80, 1.54)	0.537
Location	Right colon	64		Reference	
	Left colon and rectum	126		0.80 (0.56, 1.14)	0.213
	Appendix, small intestine	6		0.48 (0.16, 1.47)	0.200
Sync	No	61		Reference	
	Yes	135		0.76 (0.51, 1.13)	0.174
OP	Primary resected at baseline	88		Reference	
	Primary in situ at baseline	108		0.83 (0.57, 1.23)	0.357
PS	0	133		Reference	
	1	63		1.63 (1.13, 2.36)	0.009
Mets		196		1.33 (1.10, 1.62)	0.004

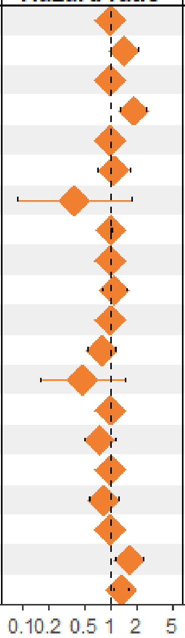

Supplementary Figure S3 Hazard ratios (HR) were calculated by multivariate Cox proportional-hazards analysis stratified by line of palliative chemotherapy. (Total_drugs = total drugs in treatment regimen, Location = primary tumor location, Sync = synchronous metastatic disease, OP = primary tumor resection, PS = performance status, Mets = number of metastatic sites).
